# Hitzeschutz im Fokus der hessischen Betreuungs- und Pflegeaufsicht: Beratung und Prüfung stationärer Einrichtungen seit dem Jahr 2004

**DOI:** 10.1007/s00103-024-03845-1

**Published:** 2024-04-19

**Authors:** Debora Janson, Henny Annette Grewe, Johanna Nickl, Laura Hannemann

**Affiliations:** 1 Deutsches Krankenhausinstitut, Düsseldorf, Deutschland; 2 Public Health Zentrum Fulda, Hochschule Fulda, Fulda, Deutschland; 3 Schule für Pflegeberufe, Städtisches Klinikum Lüneburg, Lüneburg, Deutschland; 4 GKV-Bündnis für Gesundheit in Bayern, München, Deutschland; 5 Geschäftsstelle Gesundheitsregion Plus im Landkreis Dachau, Dachau, Deutschland

**Keywords:** Gesundheitsschutz, Hitzeaktionsplan, Qualitätsmanagement, Klimawandel, Heimaufsicht, Health risks of extreme heat, Heat health action plan, Quality management, Climate change, Health authorities

## Abstract

**Hintergrund und Fragestellung:**

Hitzeextreme sind mit erheblichen gesundheitlichen Risiken verbunden, insbesondere für vulnerable Gruppen. Um diesen entgegenzuwirken, zielen gesundheitspolitische Forderungen darauf ab, Schutzmaßnahmen verpflichtend an Hitzewarnungen zu koppeln. Derartige Kopplungen existieren in Deutschland in der Regel nicht, eine Ausnahme stellen die seit 2004 umgesetzten Hitzeprüfungen und Hitzeberatungen der hessischen Betreuungs- und Pflegeaufsicht dar.

Ziele dieser Arbeit waren, die Strukturen und Abläufe der hessischen Hitzeprüfungen und Hitzeberatungen zu erfassen und Erkenntnisse für den akuten Hitzeschutz in stationären Gesundheitseinrichtungen abzuleiten.

**Methoden:**

Durchgeführt wurden 14 qualitative, leitfadengestützte Interviews mit Expert*innen der hessischen Betreuungs- und Pflegeaufsicht und mit Leitungen stationärer Einrichtungen der Altenpflege und -betreuung. Die Analyse der Interviewprotokolle erfolgte mit der inhaltlich strukturierenden qualitativen Inhaltsanalyse. Ergänzend wurden Dokumente der Aufsichtsbehörde analysiert.

**Ergebnisse:**

In den ca. 2500 stationären Einrichtungen in Hessen werden jährlich bis zu 370 Prüfungen zum Hitzeschutz durchgeführt. Diese werden entweder in bereits geplante Prüfungen integriert oder gesondert abgenommen, sie fokussieren sich auf präventive und akute Maßnahmen. Hitzeschutz lässt sich prinzipiell gut im Alltag stationärer Einrichtungen einbinden. Hohe Personalfluktuation und Ressourcenmangel stellen Herausforderungen dar.

**Diskussion:**

Prüfungen und Beratungen zum Hitzeschutz sensibilisieren für Hitzerisiken und unterstützen die Etablierung präventiver Maßnahmen. Das hessische System eignet sich zur Orientierung für andere Bundesländer.

## Abstract (en)

**Background and aims:**

Heat extremes are associated with considerable health risks, especially for vulnerable groups. To counteract these risks, public health policy calls for protective measures to be linked to heat warnings. Such links do not generally exist in Germany, with the exception of the heat inspections and consultations carried out by the Hessian health authorities since 2004.

The aims of this work were to identify the structures and processes of the Hessian heat inspections and heat consultations and to derive findings for acute response to heat in residential care and nursing facilities.

**Methods:**

We conducted 14 qualitative, semi-structured interviews with experts from the Hessian health authorities as well as with managers of residential care and nursing facilities. The analysis of the interview protocols was carried out using content-structuring qualitative content analysis. In addition, documents from the supervisory authority were analyzed.

**Results:**

Every year, up to 370 heat inspections are carried out in the approximately 2500 inpatient facilities in Hesse. They are either integrated into already planned inspections or carried out separately; they focus on preventive and acute measures. In principle, heat protection can be easily integrated into the daily routine of residential health facilities. High staff turnover and lack of resources pose challenges.

**Discussion:**

Inspections and consultations on heat management raise awareness of hot weather health risks and support the establishment of preventive measures. The Hessian system is a suitable orientation for other federal states.

## Hintergrund und Fragestellung

Im Sommer 2023 kündigte das Bundesministerium für Gesundheit (BMG) einen nationalen Hitzeschutzplan an und forderte die Länder auf, zu prüfen, „ob die Warnstufen des [Deutschen Wetterdienstes] DWD mit der Durchführung von Akutmaßnahmen verpflichtend gekoppelt werden können (z. B. Maßnahmen in stationären Pflegeeinrichtungen)“ [1]. Bisher existieren derartige Kopplungen in Deutschland nur vereinzelt, zumeist auf der Ebene von Kommunen oder Institutionen [2]. Eine Ausnahme auf der Landesebene stellen die Hitzeprüfungen und Hitzeberatungen der hessischen Betreuungs- und Pflegeaufsicht dar. Seit 2004 werden stationäre Pflege- und Betreuungseinrichtungen von der hessischen Betreuungs- und Pflegeaufsicht zur Prävention hitzebedingter Gesundheitsstörungen beraten, zu Maßnahmen angehalten und hinsichtlich deren Umsetzung während Hitzeperioden geprüft.

Die gesundheitlichen Risiken, die von sommerlichen Hitzewellen ausgehen, sind beträchtlich. Hitze hat negative und mitunter lebensbedrohliche Auswirkungen auf eine Vielzahl von Vorerkrankungen wie Herz-Kreislauf‑, Nieren- oder Lungenerkrankungen [3]. Hochrechnungen gehen davon aus, dass in Deutschland im Sommer 2003 etwa 9500 Todesfälle auf Hitzeextreme zurückzuführen waren [4]. Auch für die besondere Vulnerabilität von Einrichtungen der Altenpflege gibt es Belege. So wurde für Frankfurt am Main für die Hitzeperiode vom 03.08. bis zum 14.08.2003 eine Übersterblichkeit von etwa 200 Personen berechnet, ca. 100 der Verstorbenen lebten in Altenheimen [5].

Eine Bund-Länder-Arbeitsgruppe veröffentlichte 2017 erstmals Empfehlungen, in denen Länder und Kommunen angehalten werden, umfassende Maßnahmen zum Schutz vor hitzebedingten Gesundheitsschäden in Hitzeaktionsplänen festzuhalten [6]. Die Gesundheitsminister*innen der Länder bekräftigten diese Empfehlungen im September 2020 [7] und die Sozial- und Arbeitsminister*innen hoben im November 2020 hervor, dass „die Herausforderungen im Bereich der Pflege [dabei] besonderer Beachtung“ bedürften [8]. Seitens des Bundesministeriums für Umwelt, Naturschutz, nukleare Sicherheit und Verbraucherschutz (BMUV) wurde 2020 ein Förderprogramm für „Klimaanpassung in sozialen Einrichtungen“ aufgelegt [9], welches Pflege- und Betreuungseinrichtungen einschließt. In der Regel beschreiben Vertreter*innen der Bundesebene jedoch, dass die Verantwortung für den Hitzeschutz bei Ländern und Kommunen liege. Im August 2022 verwies die parlamentarische Staatssekretärin des BMG auf die Frage eines Abgeordneten, wie viele Pflegeeinrichtungen über kühlbare Innenräume zum Schutz vor Hitze verfügten, auf die Zuständigkeit der Länder. Auf die Frage, wie die Bundesregierung die Umsetzung der Handlungsempfehlungen für Hitzeaktionspläne einschätzen würde, antwortete der parlamentarische Staatsekretär des BMUV in ähnlicher Weise: Es sei nicht die Aufgabe des Bundes, die Umsetzung in den Ländern und Kommunen zu prüfen oder zu bewerten [10].

Die jüngsten Ankündigungen des BMG scheinen dementgegen den Bund als Akteur des Hitzeschutzes hervorzuheben. Das Impulspapier des Ministeriums schränkt allerdings ein, dass ein Hitzeschutzplan „unter Beachtung der föderalen Zuständigkeiten“ entwickelt werden solle [1]. Die Frage, ob und wie zukünftig Akutmaßnahmen zum Schutz vulnerabler Gruppen verpflichtend an die Hitzewarnungen des DWD gekoppelt werden können, lenkt den Blick auf bestehende Praxisbeispiele. In Hessen existiert eine landesweite Verbindlichkeit zur Durchführung von Hitzeschutzmaßnahmen in stationären Pflege- und Betreuungseinrichtungen bereits seit 19 Jahren und erreicht mit den Bewohnenden dieser Einrichtungen eine sehr große Anzahl von gegenüber Hitze vulnerablen Menschen. Obwohl die Hitzeprüfungen und -beratungen der hessischen Betreuungs- und Pflegeaufsicht Pioniercharakter haben, wurden bisher erst wenige Details zu deren Inhalten und Abläufen veröffentlicht. Die vorliegende Arbeit zielt darauf ab, die Strukturen der hessischen Hitzeprüfungen und -beratungen zu erfassen, deren Auswirkungen zu untersuchen und allgemeingültige Erkenntnisse für den akuten Hitzeschutz in stationären Einrichtungen abzuleiten.

## Vorgehen

Im Mittelpunkt der Arbeit stehen 14 qualitative, leitfadengestützte Interviews mit 21 Expert*innen aus verschiedenen, für die hessischen Hitzeprüfungen und -beratungen relevanten Institutionen. Die Interviews fanden online oder telefonisch mit 1–4 Teilnehmenden im Zeitraum von März 2021 bis April 2022 statt. Die Länge der Interviews betrug 50–150 min. 2 Interviews wurden mit Vertreter*innen der oberen Aufsichtsbehörde für hessische Betreuungs- und Pflegeeinrichtungen geführt. 6 Interviews fanden mit Prüfenden aus den 6 hessischen Versorgungsämtern statt, weitere 6 mit Leitungen stationärer Betreuungs- und Pflegeeinrichtungen. In 5 Fällen nahmen die Leitungen der Einrichtungen am Interview teil, in einem Fall die Pflegedienstleitung. Tab. 1 bietet eine Übersicht über die geführten Interviews.

**Table 1:** 

Institution	Aktivität zum Thema Hitze	Anzahl Interviews	Anzahl Interviewteilnehmende
Obere Aufsichtsbehörde Hessische Betreuungs- und Pflegeaufsicht	Konzeption/Sicherstellung Hitzeprüfungen/-beratungen	2	2
Ausführende Aufsichtsbehörde Amt für Versorgung und Soziales (Versorgungsamt)	Umsetzung von Hitzeprüfungen/-beratungen	6	12
Stationäre Betreuungs- und Pflegeeinrichtung	Umsetzung von Hitzeschutzmaßnahmen	6	7

Die Akquise der Interviewpartner*innen fand telefonisch und per E‑Mail statt. Zur oberen Aufsichtsbehörde bestand bereits aus früheren Projekten Kontakt. Um Expert*innen aus stationären Einrichtungen der Altenpflege und -betreuung zu gewinnen, wurden Anfragen an einen Pool aus bestehenden Kontakten des Fachbereiches Gesundheitswissenschaften der Hochschule Fulda versandt. In einem Fall wurde von einem Versorgungsamt der Kontakt zu einer Einrichtung hergestellt. Die regionale Verteilung der Einrichtungen wurde aufgrund der unterschiedlichen Zuständigkeiten berücksichtigt; die 6 interviewten Einrichtungen fielen in die Zuständigkeit von 4 der 6 Versorgungsämter.

Für die 3 Ebenen (obere Aufsichtsbehörde, ausführende Aufsichtsbehörde, Einrichtung) wurden jeweils teilstrukturierte Interviewleitfäden entwickelt. Die Interviews wurden aufgezeichnet und anschließend protokolliert. Die Interviewteilnehmenden erhielten die Erinnerungsprotokolle zur Durchsicht und Korrektur. Die Protokolle wurden anschließend mittels der inhaltlich strukturierenden qualitativen Inhaltsanalyse analysiert [11]. Nach einer Phase der initiierenden Textarbeit wurde in einem mehrstufigen Verfahren durch die Bildung von deduktiven sowie induktiven Kategorien eine inhaltliche Strukturierung der Daten erzeugt.

Eine weitere Datenquelle stellten veröffentlichte oder zur Verfügung gestellte Dokumente der hessischen Pflege- und Betreuungsaufsicht dar. Zur Verfügung gestellt wurden u. a. der Prüfleitfaden für Hitzeprüfungen sowie interne Statistiken zu Prüftätigkeiten und Einrichtungsarten. Diese wurden der Anzahl der Hitzewarntage in Hessen gegenübergestellt, welche auf der Homepage des DWD abgerufen wurden [12].

## Ergebnisse

### Entstehungsgeschichte der Prüfungen und Beratungen zum Hitzeschutz

Zunächst werden Grundlagen und Chronologie der Hitzeprüfungen und -beratungen der hessischen Betreuungs- und Pflegeaufsicht anhand der Schilderungen der Interviewpartner*innen vorgestellt. Ergänzend wurden hierzu Literaturrecherchen und eine Aufbereitung der internen Prüfstatistik der Aufsichtsbehörde sowie der historischen Hitzewarnungen vorgenommen.

**Figure 1:**
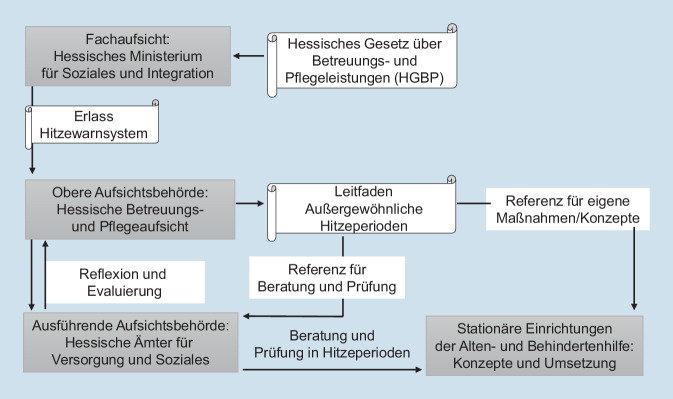

In jedem Bundesland existiert eine Heimaufsichtsbehörde, welche sicherstellt, dass Anbieter der Pflege und Betreuung die Vorgaben der jeweiligen Landesgesetze einhalten. Die Aufsichtsbehörden sind in einigen Bundesländern auf kommunaler Ebene, in anderen auf Landesebene angesiedelt [13]. Der in Hessen auf Landesebene angesiedelten oberen Aufsichtsbehörde sind 6 ausführende regionale Ämter für Versorgung und Soziales (im Folgenden Versorgungsämter) untergeordnet. Dies ist aber nicht in allen auf Landesebene angesiedelten Aufsichtsbehörden der Fall: In Sachsen prüft beispielsweise eine einzelne Behörde landesweit. Nach Einschätzung von Expert*innen aus den Aufsichtsbehörden trug die Ansiedlung der hessischen Betreuungs- und Pflegeaufsicht auf Landesebene zur schnellen und erfolgreichen Etablierung der Hitzeberatungen und -prüfungen bei, da bei kommunaler Anbindung der Heimaufsicht zusätzliche Hürden entständen, u. a. weil die Ausrichtung und Ausstattung kommunaler Aufsichtsbehörden sehr heterogen ausfallen können. Die hessische Betreuungs- und Pflegeaufsicht ist die obere Aufsichtsbehörde und war bis 2023 beim Regierungspräsidium Gießen angesiedelt. Seit dem 01.01.2023 gehört sie zu dem neu geschaffenen Hessischen Landesamt für Gesundheit und Pflege. Die Fachaufsicht liegt beim hessischen Ministerium für Soziales und Integration. Abb. 1 bietet eine Übersicht über die Institutionen und ihre Rolle bezüglich des Hitzeschutzes, in Infobox 1 ist eine Chronologie der hessischen Hitzeprüfungen und -beratungen dargestellt.

Das Engagement der hessischen Betreuungs- und Pflegeaufsicht im Hitzeschutz geht auf das außergewöhnlich heiße Jahr 2003 zurück. In einer hessischen Altenpflegeeinrichtung erregten 8 plötzliche Todesfälle im August 2003 Aufmerksamkeit und führten zu einer Landtagsdebatte über Hitzeschutz [14]. Bereits 2004 wurde eine Arbeitsgruppe zum Hitzeschutz beim hessischen Sozialministerium angesiedelt, welche die Grundlagen für die hessischen Hitzeprüfungen und -beratungen schuf und gemeinsam mit dem DWD das hessische Hitzewarnsystem entwickelte. Im gleichen Jahr begannen einige Versorgungsämter, Betreuungs- und Pflegeeinrichtungen explizit zum Thema Hitze zu prüfen und zu beraten. Die Arbeitsgruppe bereitete 2004 auch den Erlass des Sozialministeriums zum hessischen Hitzewarnsystem vor, welcher 2008 erneuert wurde. Zunächst stellte dieser sicher, dass Einrichtungen Hitzewarnungen erhalten. Neben den stationären Pflege- und Betreuungseinrichtungen adressiert der Erlass Gesundheitsbehörden, Krankenhäuser, die kassenärztliche Vereinigung und den Medizinischen Dienst der Krankenkassen. Eine verbindliche Kopplung von Maßnahmen an die Hitzewarnungen wurde nur im Bereich der Pflege- und Betreuungseinrichtungen etabliert [15]. Grundlage für sämtliche Tätigkeiten der Betreuungs- und Pflegeaufsicht ist das Hessische Gesetz über Betreuungs- und Pflegeleistungen (HGBP; [16]). Interviewpartner*innen nannten mehrere Passagen als Bezugspunkt zum Hitzeschutz. Beispielsweise werden Einrichtungen gemäß § 9 Absatz 9 verpflichtet, „eine angemessene Qualität der Betreuung einschließlich der Pflege nach dem allgemein anerkannten Stand pflegerisch-medizinischer Erkenntnisse“ anzubieten. In der Ausführungsverordnung zum HGBP ist darüber hinaus in § 12 Absatz 4 festgehalten, dass „das Raumklima, die Belichtung und die Beleuchtung einer Einrichtung“ an den Bedürfnissen der Bewohnenden auszurichten sind.

2007 veröffentlichte die hessische Betreuungs- und Pflegeaufsicht einen Leitfaden für Einrichtungen zum Umgang mit Hitzeperioden, welcher 2017 aktualisiert wurde [17]. Dieser Leitfaden legt für die Einrichtungen verbindlich fest, welche Aspekte beim Hitzeschutz beachtet werden müssen, wobei die Wahl der konkreten Maßnahmen den Einrichtungen überlassen bleibt. Die in Stichproben durchgeführten Hitzeprüfungen wurden zunächst nur von Versorgungsämtern in südhessischen Regionen durchgeführt, da diese Regionen als besonders betroffen eingeschätzt wurden. Im Jahr 2017 wurden die Hitzeprüfungen auf ganz Hessen ausgeweitet. Das System der Beratungen und Prüfungen zum Hitzeschutz wird kontinuierlich evaluiert und weiterentwickelt.

**Figure 2:**
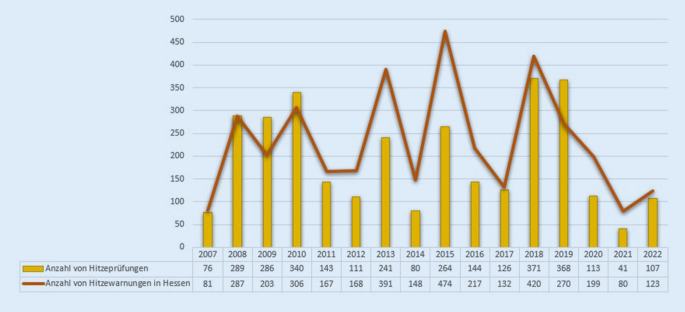

Abb. 2 zeigt die Prüftätigkeit zu Hitze seit 2007. Daten zur Anzahl der Prüfungen in früheren Jahren liegen nicht vor. 2018 erfolgten mit 371 Prüfungen bisher die meisten Hitzeprüfungen. In den Jahren 2020 und 2021 gab es aufgrund der COVID-19-Pandemie Einschränkungen und Veränderungen im Prüfgeschehen. Darüber hinaus ist die Anzahl der Hitzewarnungen des DWD abgebildet, da diese als Auslöser für die Prüfungen dienen. Dementsprechend wird in heißen Sommern häufiger geprüft. Hitzewarnungen sind allerdings Wettervorhersagen und bilden somit nicht die Anzahl tatsächlicher Hitzetage ab [18]. Der DWD weist zudem darauf hin, dass sich die Kriterien für eine Hitzewarnung mit der Zeit verändert haben [19].

### Inhalte und Abläufe der Hitzeprüfung und -beratung

Seitens der hessischen Betreuungs- und Pflegeaufsicht wurde ein Prüfleitfaden entwickelt, auf dessen Basis die 6 Versorgungsämter Hitzeprüfungen durchführen. Der Prüfleitfaden liegt den Autor*innen dieser Arbeit vor und wird in Tab. 2 zusammengefasst. Mittelfristige und kurzfristige Maßnahmen stehen im Vordergrund der Hitzeprüfungen, die während akuter Hitzeperioden stattfinden.

**Table 2:** 

Pflege	Management & Dokumentation	Raumkühlung
– Vulnerable Bewohnende sind identifiziert und Gesundheitszustand wird häufiger kontrolliert – Besonderes Augenmerk liegt auf den Anzeichen einer Exsikkose – Verstärktes Kontrollieren der Körpertemperaturen – Medikamente sind bezüglich hitzebedingter Veränderungen angepasst	– DWD-Newsletter ist abonniert – Konzept zu Hitze ist vorhanden – Mitarbeitende kennen Konzept und sind über Hitzewarnung informiert – Hitzeperiode ist in Einsatzplanung berücksichtigt – Temperaturen sind in ausgewählten Räumen gemessen und dokumentiert – Trinkprotokolle sind bei Bedarf angelegt – Ausgewählte pflegerische Maßnahmen sind dokumentiert	– Maßnahmen zur Kühlung sind umgesetzt (Außenbeschattung, Jalousien, Klimageräte, Hitzeschutzfolie) – Lüftung der Räume ist an Hitze angepasst (i. d. R. Nachtlüftung) – Medikamente sind kühl gelagert
**Aktivität, Kleidung, Bettwäsche**		**Speisen & Getränke**
– Kleidung ist an Hitze angepasst – Bettwäsche ist an Hitze angepasst – Auf körperlich anstrengende Freizeitangebote wird verzichtet		– Hitzeangepasster Speiseplan – Größeres Angebot von Zwischenmahlzeiten und Getränken – Flüssigkeitsaufnahme wird verstärkt unterstützt

Insgesamt beschrieben die interviewten Einrichtungen, dass die Umsetzung des Hitzemanagements gut in den Alltag integrierbar sei und dass dies mit großer Selbstverständlichkeit erfolge. Die konkrete Umsetzung der Maßnahmen fällt sehr individuell aus und ist von den lokalen Gegebenheiten geprägt. Beispielsweise berichteten alle interviewten Einrichtungen, dass körperlich anstrengende Freizeitbeschäftigungen während Hitze ausfallen oder angepasst werden. In einem Fall wurde beschrieben, dass Angebote in kühle Kellerräume verlegt würden. Eine andere Einrichtung setzte auf die Erhöhung gemeinsamer Trinkrunden bei Gruppenaktivitäten, während in einer weiteren Einrichtung das Programm der Sozialarbeit von Gruppenangeboten hin zu individuellen Besuchen in den Privaträumen geändert wird. Entsprechend der großen Heterogenität der Einrichtungen ist sowohl in der Beratung als auch in den Prüfungen der Betreuungs- und Pflegeaufsicht Offenheit hinsichtlich der gewählten Umsetzung vorgesehen.

Langfristige bauliche Maßnahmen werden von der Betreuungs- und Pflegeaufsicht auch unabhängig von den Temperaturen z. B. bei Neubauanträgen beratend angesprochen. Hinsichtlich der baulichen Maßnahmen schilderten mehrere Interviewteilnehmende aus Aufsichtsbehörden sowie aus den Einrichtungen Handlungsbedarf. Es wurden finanzielle und gesetzliche Hürden beschrieben, beispielsweise könnten Anforderungen des Denkmalschutzes der Hitzeanpassung eines Gebäudes entgegenstehen. Auch wünschten sich mehrere Personen, dass Hitzeschutz bei Neubauten mit größerer Verbindlichkeit in den Gesetzen festgelegt werden sollte.

**Figure 3:**
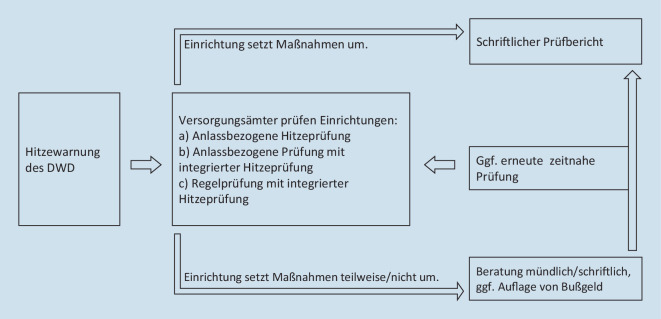

Aus den Interviews mit den Aufsichtsbehörden wurden typische Abläufe zur Beratung und Prüfung zum Hitzeschutz rekonstruiert. Diese werden im Folgendem erläutert und sind auch in Abb. 3 dargestellt.

Auslöser für Hitzeprüfungen der hessischen Betreuungs- und Pflegeaufsicht sind die vom DWD auf Landkreisebene herausgegeben Hitzewarnungen. Prüfungen zu Hitze werden bei beiden möglichen Warnstufen, Stufe 1 „starke Wärmebelastung“ und Stufe 2 „extreme Wärmebelastung“ [18] vorgenommen. Erhält ein Versorgungsamt eine Hitzewarnung für einen Landkreis in seiner Zuständigkeit, beginnt die Planung der Hitzeprüfungen.

Die 6 Versorgungsämter nennen unterschiedliche Kriterien, nach denen sie Einrichtungen auswählen. Einigkeit bestand hinsichtlich der Einrichtungsart: Alle Versorgungsämter fokussieren sich hauptsächlich auf Einrichtungen der Altenhilfe, da hier die Bewohnenden als besonders vulnerabel für hitzebedingte Gesundheitsschäden eingeschätzt werden. Dies spiegelt sich in der internen Statistik zu den Prüfungen wider: 84 der 107 Prüfungen im Jahr 2022 wurden in Alten- und Pflegeheimen durchgeführt, die weiteren Hitzeprüfungen verteilten sich auf andere Einrichtungsarten (12 in Tagespflegeeinrichtungen, 10 in besonderen Wohnformen der Behindertenhilfe, eine in betreuter Wohnform der Behindertenhilfe). Weitere Auswahlkriterien, die die Versorgungsämter mit unterschiedlicher Gewichtung berücksichtigen, betreffen die Fragen, ob die Einrichtung aufgrund ihrer Lage Hitze besonders ausgesetzt ist, ob es in einer vorherigen Prüfung Auffälligkeiten gab und wie lange diese zurückliegt oder ob die Prüfungen mehrerer Einrichtungen aufgrund von örtlicher Nähe verbunden werden können.

Hitzeprüfungen werden auf 3 Weisen durchgeführt, entweder als a) anlassbezogene Hitzeprüfung, b) anlassbezogene Prüfung mit integrierter Hitzeprüfung oder c) als Regelprüfung mit integrierter Hitzeprüfung. Anlassbezogene Prüfungen werden vorgenommen, wenn ein konkreter Anlass besteht, beispielsweise die Beschwerde eines Angehörigen. Anlassbezogene Hitzeprüfungen (a) fokussieren sich ausschließlich auf Hitzeschutzmaßnahmen, bei ihnen gilt die Hitze selbst als Anlass zur Prüfung. Regelprüfungen werden hingegen kontinuierlich durchgeführt, um sicherzustellen, dass die Einrichtungen alle gesetzlichen Anforderungen erfüllen. Regelprüfungen sind deutlich aufwendiger, da sie sämtliche Kriterien des HGBP [16] abdecken. Liegen Hitzewarnungen vor, können Versorgungsämter die für Hitze relevanten Fragen in bereits geplante Prüfungen integrieren (b oder c).

Bei anlassbezogenen Hitzeprüfungen handelt es sich um zusätzliche Prüfungen, die somit zusätzliche Ressourcen des jeweiligen Versorgungsamtes binden, während der Mehraufwand von Hitzeprüfungen, die mit bereits geplanten Prüfungen verbunden werden, überschaubar bleibt. Allerdings kann nur mit anlassbezogenen Hitzeprüfungen eine relativ große Anzahl von Einrichtungen in einer Hitzeperiode erreicht werden, da diese einen geringeren Zeitaufwand benötigen. Die prinzipiellen Prüfungsabläufe sind von der oberen Aufsichtsbehörde gesetzt, jedoch verfügen die Versorgungsämter über Gestaltungsspielraum, der auch die Wahl der Prüfungsform betrifft. Während einige Versorgungsämter angaben, selten oder nie anlassbezogene Hitzeprüfungen durchzuführen, beschrieben andere, dass während Hitzeperioden bereits geplante Prüfungen zugunsten dieser verschoben werden.

Im Anschluss an eine Prüfung wird einer Einrichtung mitgeteilt, wie weit ihr Hitzemanagement den gesetzlichen Anforderungen entspricht. Wird in der Hitzeprüfung ersichtlich, dass Maßnahmen nicht ausreichend oder fehlerhaft umgesetzt werden, folgt eine fachliche Mängelberatung. Die Prüfenden agieren hier situationsangepasst und nehmen die Mängelberatungen schriftlich oder mündlich, mit oder ohne Aufforderung einer Bußgeldzahlung vor. Kleinere Mängel können während der Anwesenheit der Prüfenden direkt behoben werden. Zumeist folgt auf eine Mängelfeststellung zeitnah eine erneute Prüfung, teilweise am selben Tag. Als letzte Konsequenz könnte die Aufsichtsbehörde die Schließung einer Einrichtung veranlassen, was anlässlich eines unzureichenden Hitzeschutzes in Hessen aber noch nie vorgekommen ist. Expert*innen führen dies darauf zurück, dass sich die Mängel im Hitzeschutz mit verhältnismäßig wenig Aufwand beheben ließen.

#### Interaktion zwischen Einrichtungen und Aufsichtsbehörde

Insgesamt betonten Prüfende ihr lösungsorientiertes Vorgehen, bei dem die Unterstützung der Einrichtungen im Vordergrund steht. Sie möchten mit den Einrichtungen auf Augenhöhe zusammenarbeiten, Erfahrungen weitergeben und individuelle Lösungen finden. Im Rundgang durch die Einrichtung wird der Dialog zu Mitarbeitenden und Bewohnenden gesucht. Auf die Nachfrage, ob den Bewohnenden ausreichend Getränke zur Verfügung stehen, verzichten einige Prüfende bewusst. Dies gelte in der Regel als selbstverständlich und Erkenntnisse hierzu ließen sich gut durch Beobachtung gewinnen. Dieser Aspekt verdeutlicht die Gratwanderung, die vorgenommen wird, um in einer Prüfung als Beratungsinstanz aufzutreten.

Die Einrichtungen beschrieben unterschiedliche Erfahrungen mit Hitzeprüfungen. Einige schilderten diese als zusätzliche Belastung oder hatten den Eindruck, ihre individuelle Situation sei nicht ausreichend berücksichtig worden. Andere zeigten sich dankbar für den Blick von außen. Hitzeprüfungen und -beratung sind zumeist miteinander verbunden. In den Interviews fiel auf, dass seitens der Versorgungsämter häufig der Beratungsaspekt betont wurde, während für die Einrichtungen der Prüfungscharakter im Vordergrund zu stehen schien. Hitzeberatungen finden aber nicht ausschließlich eingebunden in Prüfungen statt. Einrichtungen wenden sich auch jenseits dieser mit Beratungsanfragen zum Hitzeschutz an die Versorgungsämter.

Für bestimmte Aspekte stellen die Träger der Einrichtungen die Adressaten der Aufsichtsbehörden dar. Dies betrifft beispielsweise bauliche Elemente, da hier die Anpassungsmöglichkeiten innerhalb der Einrichtung selbst begrenzt sind, sowie Aspekte, die mehrere von einem Träger betriebene Einrichtungen betreffen können.

### Wirkung der Prüfung

Die interviewten Einrichtungen vermittelten insgesamt den Eindruck, dass sie ihr Hitzemanagement mit großer Selbstverständlichkeit umsetzten. Alle beschrieben, dass sie ein Konzept zum Hitzeschutz erarbeitet haben und dieses bei Hitze umsetzten. In 3 Interviews hieß es, dass das Hitzeschutzkonzept beim zuständigen Versorgungsamt vorgelegt worden sei. Jedoch beschrieben auch mehrere Interviewpartner*innen, dass häufiger Personalwechsel in Einrichtungen dazu führen könne, dass Wissen oder etablierte Qualitätsstandards verloren gingen.

Einzelne Prüfende berichteten von kritischen Situationen während Hitzeprüfungen, die nach akutem Handlungsbedarf verlangten. Die Gesundheit von Bewohnenden war durch überhitzte Zimmer ernsthaft gefährdet und eine sofortige Raumkühlung oder die Verlegung der Person wurde von den jeweiligen Prüfenden begleitet. Diese Situationen stellten allerdings Ausnahmen in langjährigen Prüftätigkeiten dar. Von der oberen Aufsichtsbehörde wurde als Indikator für die Wirksamkeit der Hitzeprüfungen angeführt, dass in jenen Regionen, die erst seit 2017 verstärkt Hitzeprüfungen vornähmen, deutlich mehr Mängel festgestellt würden als in jenen, die hier bereits in den frühen 2000er-Jahren aktiv waren.

Eine Einrichtungsleitung erläuterte, dass die Branche der Pflegeheime recht traditionell sei und dass genau beobachtet würde, welche Themen Institutionen, wie die Betreuungs- und Pflegeaufsicht, priorisierten. Einrichtungen reagierten hierauf und in zugehörigen Netzwerken würden Diskussionen angestoßen*.*

## Diskussion

Der Klimawandel führt weltweit zu zunehmenden Hitzewellen [20], gleichzeitig wachsen in Deutschland durch die Alterung der Bevölkerung die Anzahl pflegebedürftiger Personen [21] und damit die Gruppe der besonders von Hitze gefährdeten Menschen. Allein für den Sommer 2022 gehen Hochrechnungen von über 8000 hitzebedingten Todesfällen in Deutschland aus [22]. Daraus ergibt sich die Notwendigkeit, bundesweit verbindliche Strukturen zur Prävention hitzebedingter Morbidität und Mortalität zu schaffen.

Das Land Hessen setzt mittels Beratungen und Prüfungen der Betreuungs- und Pflegeaufsicht Standards zum Hitzeschutz. Mehre Faktoren konnten in der Analyse als begünstigend dafür identifiziert werden, dass in Hessen die langfristige Etablierung eines Systems zum Hitzeschutz bereits Anfang der 2000er-Jahre gelang. Zunächst schockierten die hitzebedingten Todesfälle im Sommer 2003 die hessische Politik und Gesellschaft. Der Fall eines Altenheims, in dem es innerhalb weniger Tage zu vielen Todesfällen gekommen war, erhielt große mediale Aufmerksamkeit. Die zu diesem Zeitpunkt vorhandene Sensibilisierung für Hitzerisiken ging mit Handlungswillen von Entscheidungstragenden einher. Einzelne engagierte Personen in den Aufsichtsbehörden, denen die Errichtung dieses Systems ein Anliegen war, spielten dabei eine entscheidende Rolle. Auch die Ansiedlung der Behörde auf Landesebene wurde als Vorteil für ein derartiges System gesehen.

Auch wenn sich der Aufbau und die Ansiedlung der Heimaufsichten in den Bundesländern unterscheiden, lassen sich grundsätzliche Erkenntnisse aus Hessen auf andere Länder übertragen. Im Bereich des Heimrechts liegt die Gesetzgebungskompetenz bei den Ländern, die jeweils über eigene Heimgesetze verfügen [13, 23]. Im HGBP ist „Hitze“ nicht explizit erwähnt, dennoch konnten ausreichend Bezüge zur Rechtfertigung der Hitzeprüfungen gefunden werden. Der in Hessen bemühte Abschnitt (§ 9 Absatz 9 des HGBP) ist beinah deckungsgleich in den Heimgesetzen anderer Länder enthalten (vergleiche z. B. Niedersächsisches Gesetz über unterstützende Wohnformen (NuWG) § 11.1 oder Wohn‑, Teilhabe- und Pflegegesetz (WTPG) § 10.2; [16, 23]). Neben gesetzlichen Grundlagen gehört der politische Wille der Landesregierung zu den notwendigen Voraussetzungen für die Etablierung von Hitzeschutzmaßnahmen. In Hessen wurde dieser bereits vor der Veröffentlichung des landesweiten Hitzeaktionsplans im Jahr 2023 in Form eines Erlasses des Sozialministeriums geäußert.

In der hier vorgestellten Studie wurde zudem deutlich, dass es stationären Pflege- und Betreuungseinrichtungen prinzipiell nicht an Informationen oder Empfehlungen zum Umgang mit Hitze mangelt. Damit diese Wirkung entfalten, ist die Einbindung in ein Qualitätsmanagementsystem mit verbindlichen Strukturen notwendig, welches auch dem Verlust der Standards durch Herausforderungen wie eine hohe Personalfluktuation entgegenwirken kann. Die Einbindung einer externen Instanz kann sicherstellen, dass das Thema nicht aufgrund geringer Risikowahrnehmung oder Saisonalität in Vergessenheit gerät. Insgesamt scheint die regelmäßige Präsenz der Prüfenden zu einem routinierten Umgang der hessischen Einrichtungen in der Umsetzung von Hitzeschutzmaßnahmen beizutragen.

Prüfung und Beratung zum Hitzeschutz sind in Hessen eng miteinander verbunden und verfolgen das Ziel einer schnellen und praxisorientierten Lösungsfindung. Eine Konsequenz hieraus sind nur in Teilen standardisierte Prozesse, ein Aspekt, der auch durch die relative Autonomie der 6 Versorgungsämter bedingt ist. Dies schränkt die Aussagekraft der von den Prüfenden in die Prüfstatistik des Landes eingespeisten Daten zwar ein, lässt jedoch den Spielraum, im Prüf- und Beratungsprozess auf die Individualität der Einrichtungen einzugehen. Hinsichtlich der Beratung stellt sich die Frage, ob Einrichtungen diese intensiver oder unbefangener in Anspruch nehmen würden, wenn sie von einer Instanz angeboten würde, die in einem hierarchiefreien Verhältnis zu ihnen stünde. Allerdings bleibt auch offen, welche unabhängige Institution kompetente, kostenlose Beratung zum Hitzeschutz speziell für diese Branche anbieten könnte.

Für das Hitzemanagement der stationären Einrichtungen in Hessen stellt das Land keine zusätzlichen Ressourcen bereit. Viele Hitzeschutzmaßnahmen können ohne zusätzliche Investitionen umgesetzt werden, da sie primär eine Veränderung der Abläufe darstellen. Jedoch sind einige Maßnahmen, wie häufigeres Wechseln der Bettwäsche, höhere Getränkeausgaben oder zusätzliche pflegerische Maßnahmen wie Fiebermessen mit einem Mehraufwand für die Einrichtungen verbunden. Dabei leidet die Branche unter dem Pflegenotstand. Für Einrichtungen, deren Stellen nicht voll besetzt sind, ist die Umsetzung selbst einer nur kleinen zusätzlichen Aufgabe eine große Herausforderung. Auch für die Aufsichtsbehörden bedeuten Prüfungen bei Hitze Mehraufwand, insbesondere wenn diese nicht nur in bereits geplante Prüfungen integriert, sondern gesondert durchgeführt werden. Hieraus lässt sich die Empfehlung für Bund und Länder ableiten, dass Hitzeschutz in stationären Pflege- und Betreuungseinrichtungen mit Ressourcen unterlegt werden sollte. Ob die Förderung von Klimaanpassung in sozialen Einrichtungen durch das BMUV [9] für eine flächendeckende Unterstützung der mehr als 16.000 stationären Pflegeeinrichtungen in Deutschland [24] ausreicht, ist zweifelhaft, da insbesondere bauliche Maßnahmen hohe Investitionen bedeuten und einzelne Einrichtungen leicht überfordern. Ebenso bedeutsam und deutlich kostengünstiger gegenüber einer nachträglichen Sanierung ist die Beachtung von Hitzeschutz bei Neubauten.

## Fazit

Prüfungen und Beratungen zum Hitzeschutz in Betreuungs- und Pflegeeinrichtungen tragen zur Sensibilisierung für Hitzerisiken bei und unterstützen die Etablierung präventiver Maßnahmen. Bundesländer, die an der Etablierung von Hitzeschutzmaßnahmen arbeiten, können sich am mehrjährig praktizierten System der Hitzeprüfungen und -beratungen des Land Hessens orientieren und ausgewählte Strukturen übertragen. Hitzeschutz sollte mit Ressourcen unterlegt werden. Das betrifft besonders die ressourcenintensive Gebäudesanierung.

### Infobox Chronologie zu hessischen Hitzeprüfungen in stationären Pflege- und Betreuungseinrichtungen (eigene Darstellung)

**2003**: Hitzesommer in Europa, Hessen gehört zu den stark betroffenen Regionen

**2004**: Gründung einer Arbeitsgruppe zum Thema Hitzeschutz auf Landesebene, Erlass des hessischen Sozialministeriums zum Hitzewarnsystem, erste Hitzeprüfungen in Einrichtungen der Alten- und Behindertenpflege

**2005**: Die in Hessen erprobten Hitzewarnungen des Deutschen Wetterdienstes (DWD) werden auf die Bundesrepublik ausgeweitet

**2007**: Hessische Betreuungs- und Pflegeaufsicht gibt eigenen Leitfaden für Einrichtungen zum Umgang mit Hitze heraus

**2013**: Umstellung des DWD-Hitzewarnsystems zu einem Hitze-Newsletter, bis dahin wurden Faxe/E-Mails von der Aufsichtsbehörde an die Einrichtungen geschickt

**2017**: Hessische Betreuungs- und Pflegeaufsicht veröffentlicht überarbeitete Version des Leitfadens für Einrichtungen und fordert weitere hessische Regionen zu verstärkten Hitzeprüfungen auf

**2020**: Aufgrund der COVID-19-Pandemie werden Hitzeprüfungen telefonisch umgesetzt, um eine zusätzliche Belastung der Einrichtungen zu vermeiden

**2021: **Prüfungen finden wieder in Präsenz statt, aber mit inzidenzabhängigen Beschränkungen

**2023**: Hessischer Hitzeaktionsplan wird vom Hessischen Ministerium für Soziales und Integration veröffentlicht, das System der Hitzeprüfungen ist hierin integriert; Hessische Betreuungs- und Pflegeaufsicht wird Teil des neu gegründeten Hessischen Landesamts für Gesundheit und Pflege
